# Mental Health Among Bus Drivers and Conductors: A Cross-Sectional Study From Karaikal, South India

**DOI:** 10.7759/cureus.43273

**Published:** 2023-08-10

**Authors:** K Mohamed Ali, K Mujibur Rahman, Gayathri S, S Nancy, S Sathish Kumar

**Affiliations:** 1 Department of Community Medicine, Vinayaka Mission’s Medical College and Hospital, Vinayaka Mission’s Research Foundation - Deemed to be University (VMRF-DU), Karaikal, IND

**Keywords:** mental health, depression, anxiety, transport workers, conductors, drivers

## Abstract

Background

Health hazards faced by bus drivers and conductors due to their stressful working conditions can vary greatly depending on the country and specific circumstances. In some regions, stringent regulations mitigate the risks, while in others, lack of enforcement exacerbates the situation. The common mental health issues faced by bus drivers and conductors are anxiety and depression. Therefore, this study was planned to determine the prevalence of anxiety and depression among bus drivers and conductors and identify the risk factors associated with depression and anxiety.

Methodology

A cross-sectional study was conducted among the bus drivers and conductors working in government and private transport in Karaikal, Puducherry, South India. After institutional ethics committee clearance, 450 male transport workers with a mean age of 42.6 ± 8.56 (SD) years were interviewed to assess their mental status according to the Hospital Anxiety and Depression Scale. Bivariate and multivariate analyses were employed using SPSS software version 20 (IBM Corp., Armonk, NY, USA) to ascertain the association between dependent (anxiety/depression) and independent variables.

Results

About 16% and 13% of bus drivers and conductors were suffering from anxiety and depression, respectively. Age, education, marital status, bus route type, years of experience, and depression were significantly associated with anxiety. Whereas type of employment, years of experience, and anxiety were significantly associated with depression (p < 0.05).

Conclusions

Anxiety and depression in drivers and conductors constitute a major public health problem. Proper job rotation, enabling good ergonomics, and social support are some recommendations to promote mental health among transport workers.

## Introduction

Bus drivers and conductors are facing various health hazards due to their stressful working conditions [1]. They are exposed to various occupational hazards which leads to deterioration of their health over a period of time [1]. Long-haul drivers have extended working hours; stressful working conditions; exposure to extremes of temperatures, noise, and vibration; and irregular sleep patterns which over a period of time affect their performance and health [2]. The common issues faced by them are sleep disturbances and stress disorders such as anxiety and depression [2].

In addition, long-haul bus drivers and conductors do not have proper boarding facilities at outstations, forcing them to sleep in buses or in cramped overcrowded places arranged by their employers [3]. These can directly affect their performance, driving skills, and health in the long run [3]. In addition to their health problems, the above conditions may also decrease their concentration levels which can lead to serious accidents, endangering their own lives and the lives of other co-passengers [4]. Hence, the prevalence of morbidities is higher in bus drivers and conductors than in the general population.

Hence, this cross-sectional study was conducted to determine the prevalence of anxiety and depression among bus drivers and conductors and identify the risk factors associated with depression and anxiety.

## Materials and methods

Study setting and participants

An analytical cross-sectional study was conducted among the bus drivers and conductors working in government and private transport in Karaikal, located on the southeast coast of India, for one year. Bus drivers drive the bus, and conductors collect fares from passengers and are expected to assist drivers in maintaining harmony among passengers. We included all those who were aged >20 years and currently employed in transport services in Karaikal divisions either as a driver or a conductor and excluded all individuals who did not provide consent for the study and those who were seriously ill. Based on the inclusion and exclusion criteria, about 52 individuals declined to participate in the study.

Sample size and sampling

Considering a prevalence of 24.28% of anxiety [1], an absolute margin of error of 4%, and a 95% confidence interval, the desired sample size was 441. The minimum sample size required to obtain a predetermined outcome of association between dependent (anxiety/depression) and independent variables for the study was 450. The sampling frame comprised the registered list of drivers and conductors, and the participants were selected by simple random sampling using random number tables.

Data collection

After obtaining informed consent, a schedule (questionnaire) was used to collect information regarding sociodemographic profile, working environment, and mental health. A pretested, standardized, semi-structured schedule was developed based on the STEPS questionnaire [5], the WHO Occupational Health Manual [6], and the Hospital Anxiety and Depression Scale (HADS) [7]. HADS is a 14-item scale with a score ranging from 0 to 3 for each item. The final scoring used in HADS was 0-7 (normal), 8-10 (borderline abnormal), and 11-21 (abnormal). The face validity of each item and content validity of each domain of the schedule was ascertained by experts. The schedule was prepared in English, translated into Tamil keeping semantic equivalence, and then back-translated into English by two language experts to check the translation.

Data analysis

Data were entered in Microsoft Excel 2010 and analyzed in SPSS software version 20.0 (IBM Corp., Armonk, NY, USA). For descriptive statistics, frequency, percentage, mean, and standard deviation (SD) were calculated. For inferential statistics, initially, bivariate analysis was performed to ascertain the relationship between dependent (anxiety/depression) and independent variables (sociodemographic profile and work experience). Subsequently, for the significant variables in bivariate analysis, multivariate logistic regression analysis (LINK FUNCTION = LOGISTIC) with various models in a nested manner was employed. P-values <0.05 were considered to reject the null hypothesis. Diagnostic tests were performed after modeling to assess the goodness-of-fit and assumptions pertaining to logistic regression.

Ethical considerations

The Institutional Research Advisory Committee reviewed the proposal, and the Institutional Ethics Committee (Vinayaka Mission’s Research Foundation Ethics Committee) approval was obtained (approval number: IEC-VMMC-04-10-2017).

## Results

Table 1 shows that a majority of the study population, 36.0%, were in the age group of 35 to 44 years. About 51.1% of the study population resided in rural areas. Nearly 41.6% had completed their higher secondary education, and 89.1% of the study participants were married. Almost 64.9% of transport workers were working in the government sector, and 72.2% were working on long-distance bus routes. About 41.3% of drivers and conductors had >15 years of work experience.

**Table 1:** Description of sociodemographic characteristics of drivers and conductors (N = 450).

Variables	Drivers	Conductors	Total
	n (%)	n (%)	n (%)
Age group (years)			
<25	2 (9.7)	12 (7.6)	14 (3.1)
25–34	55(18.8)	26 (16.6)	81 (18.0)
35–44	111 (37.9)	51 (32.5)	162 (36.0)
45–54	88 (30.0)	61 (38.9)	149 (33.1)
>54	37 (12.6)	7 (4.5)	44 (9.8)
Residence			
Urban	156 (53.2)	64 (40.8)	220 (48.9)
Rural	137 (46.8)	93 (59.2)	230 (51.1)
Education			
Primary	2 (0.7)	4 (2.5)	6 (1.3)
Secondary	79 (27.0)	49 (31.2)	128 (28.4)
Higher secondary	138 (47.1)	49 (31.2)	187 (41.6)
Graduate	74 (25.3)	55 (35.0)	129 (28.7)
Marital status			
Married	260 (88.7)	141 (89.8)	401 (89.1)
Single	24 (8.2)	16 (10.2)	40 (8.9)
Divorced	5 (1.7)	0 (0)	5 (1.1)
Separated	4 (1.4)	0 (0)	4 (0.9)
Employment			
Government	189 (64.5)	103 (65.6)	292 (64.9)
Private	104 (35.5)	54 (34.4)	158 (35.1)
Bus route type			
Mofussil	45 (15.4)	70 (44.6)	115 (25.6)
Long distance (≥200 km)	247 (84.3)	78 (49.7)	325 (72.2)
School bus	1 (0.3)	9 (5.7)	10 (2.2)
Experience (years)			
<1	65 (22.2)	51 (32.5)	116 (25.8)
1.1–5	39 (13.3)	13 (8.3)	52 (11.6)
5.1–10	23 (7.8)	17 (10.8)	40 (8.9)
10.1–15	38 (13.0)	18 (11.5)	56 (12.4)
>15	128 (43.7)	58 (36.9)	186 (41.3)

**Figure 1:**
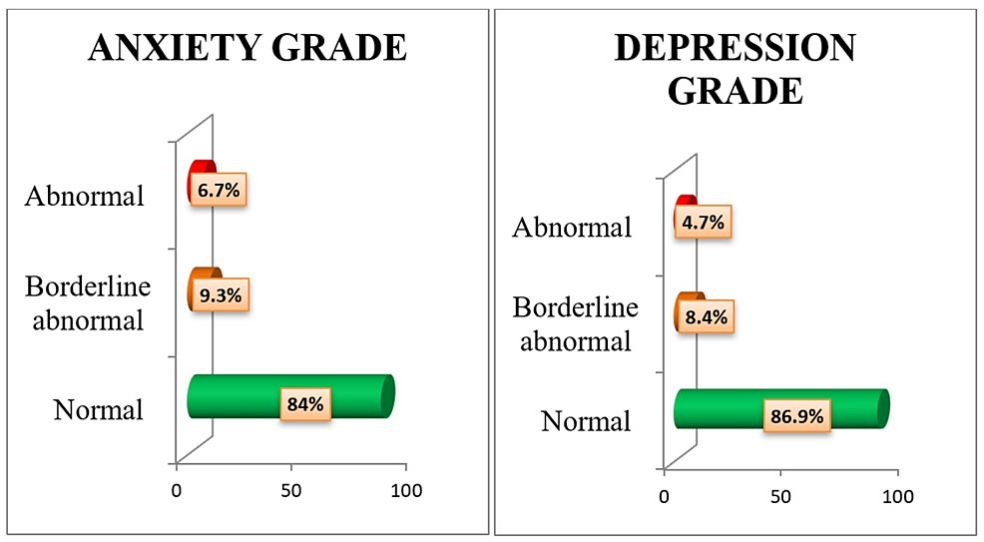
Bar diagram showing the distribution of anxiety and depression grading among drivers and conductors (N = 450).

Among the 450 transport workers, 72 (16%) and 59 (13.1%) were suffering from symptoms of anxiety and depression, respectively, according to HADS. About 21% of conductors and 13.3% of drivers had symptoms of anxiety, and 19.1% of conductors and 9.9% of drivers had symptoms of depression. Out of the 72 workers who had symptoms of anxiety, 9.3% were in the borderline state (HADS scoring = 8-10), and out of the 59 workers with symptoms of depression, 8.4% were in the borderline state (HADS scoring = 8-10) (Figure 1).

On bivariate analysis, the study population with lesser age, who were urban residents, who had completed a secondary level of education, were single, and were employed in the private sector were positively associated with anxiety, and those with >15 years of experience and worked in Mofussil and long-distance transport services were negatively associated with anxiety (p < 0.05). The presence of depression was also significantly associated with anxiety. on multivariable analysis, sociodemographic variables, such as age, education, marital status, bus route type, years of experience, and depression, were significantly associated with anxiety (p < 0.05) (Table 2).

**Table 2:** Bivariate and multivariate logistic regression models of anxiety among the study population (N = 450). In multivariate logistic regression, AOR was calculated only for the significant variables in bivariate logistic regression (crude odds ratio). *P-value <0.05; OR: odds ratio; AOR: adjusted odds ratio; CI: confidence interval; 1: reference

Independent variable	Total (n)	Anxiety, n (%)	OR (95% CI)	P-value	AOR (95% CI)	P-value
Age						
(Continuous variable)	450	72 (16.0)	0.86 (0.82–0.89)	0.001*	0.89 (0.81–0.97)	0.009*
Place of living						
Urban	220	45 (20.5)	1.93 (1.15–3.24)	0.013*	3.38 (0.99–11.46)	0.051
Rural	230	27 (11.7)	1		1	
Education						
Primary	6	0 (0)	0 (0–0)	0.999	0 (0–0)	0.999
Secondary	128	29 (22.7)	2.22 (1.12–4.39)	0.021*	102.64 (17.01–619.06)	0.001*
Higher secondary	187	28 (15.0)	1.33 (0.68–2.62)	0.395	9.52 (2.55–35.54)	0.001*
Graduate	129	15 (11.6)	1		1	
Marital status						
Single	40	15 (37.5)	3.62 (1.80–7.28)	0.001*	13.68 (2.22–84.05)	0.005*
Divorced	5	0 (0)	0 (0–0)	0.999	0 (0–0)	0.999
Separated	4	0 (0)	0 (0–0)	0.999	0 (0–0)	0.999
Married	401	57 (14.2)	1		1	
Occupation						
Conductor	157	33 (21.0)	1.73 (1.04–2.88)	0.035*	1.37 (0.46–4.01)	0.564
Driver	293	39 (13.3)	1		1	
Type of employment						
Private	158	61 (38.6)	16.06 (8.12–31.78)	0.001*	4.06 (0.89–18.46)	0.069
Government	292	11 (3.8)	1		1	
Bus route type						
Mofussil	115	21 (18.3)	0.22 (0.06–0.84)	0.027*	0.06 (0.01–0.58)	0.015*
Long distance	325	46 (14.2)	0.16 (0.05–0.59)	0.006*	0.02 (0.00–0.22)	0.001*
School bus	10	5 (50.0)	1		1	
Experience						
1–5 years	52	16 (30.8)	1.39 (0.67–2.88)	0.367	103.1 (13.57–784.73)	0.001*
6–10 years	40	13 (32.5)	1.51 (0.68–3.32)	0.302	4.31 (0.85–21.79)	0.077
11–15 years	56	9 (16.1)	0.60 (0.26–1.38)	0.231	4.80 (0.95–24.33)	0.058
>15 years	186	6 (3.2)	0.10 (0.04–0.26)	0.001*	5.69 (0.76–42.45)	0.089
<1 year	116	28 (24.1)	1		1	
Depression						
Yes	59	39 (66.1)	21.15 (11.08–40.37)	0.001*	47.74 (13.20–172.66)	0.001*
No	391	33 (8.4)	1		1	

On bivariate logistic regression, sociodemographic variables, such as age, occupation, type of employment, years of experience, and anxiety, were significantly associated with depression (p < 0.05). On multivariable logistic regression, those who were employed in the private sector were positively associated with depression, and those with >15 years of experience were negatively associated with depression. The presence of anxiety was also significantly associated with depression (p < 0.05) (Table 3).

**Table 3:** Bivariate and multivariate logistic regression models of depression among the study population (N = 450). In multivariate logistic regression, AOR was calculated only for the significant variables in bivariate logistic regression (crude odds ratio). *P-value <0.05; OR: odds ratio; AOR: adjusted odds ratio; CI: confidence interval; 1: reference

Independent variable	Total (n)	Depression, n (%)	OR (95% CI)	P-value	AOR (95% CI)	P-value
Age						
(Continuous variable)	450	59 (13.1)	0.89 (0.86–0.92)	0.001*	1.05 (0.98–1.10)	0.124
Place of living						
Urban	220	27 (12.3)	0.87 (0.50–1.50)	0.607		
Rural	230	32 (13.9)	1			
Education						
Primary	6	0 (0)	0 (0–0)	0.999		
Secondary	128	18 (14.1)	0.94 (0.47–1.90)	0.879		
Higher secondary	187	22 (11.8)	0.77 (0.39–1.49)	0.442		
Graduate	129	19 (14.7)	1			
Marital status						
Single	40	4 (10.0)	0.69 (0.23–2.04)	0.512		
Divorced	5	0 (0)	0 (0–0)	0.999		
Separated	4	0 (0)	0 (0–0)	0.999		
Married	401	55 (13.7)	1			
Occupation						
Conductor	157	30 (19.1)	2.15 (1.23–3.73)	0.007*	1.29 (0.57–2.90)	0.538
Driver	293	29 (9.9)	1		1	
Type of employment						
Private	158	49 (31.0)	12.67 (6.20–25.9)	0.001*	5.32 (1.71–16.62)	0.004*
Government	292	10 (3.4)	1		1	
Bus route type						
Mofussil	115	26 (22.6)	1.16 (0.23–5.84)	0.850		
Long distance	325	31 (9.5)	0.42 (0.09–2.07)	0.288		
School bus	10	2 (20.0)	1			
Experience						
1–5 years	52	10 (19.2)	0.62 (0.28–1.39)	0.250	0.37 (0.12–1.12)	0.079
6–10 years	40	10 (25.0)	0.87 (0.38–1.99)	0.751	0.53 (0.17–1.62)	0.263
11–15 years	56	2 (3.6)	0.10 (0.02–0.42)	0.002*	0.08 (0.01–0.4)	0.002*
>15 years	186	5 (2.7)	0.07 (0.02–0.19)	0.001*	0.21 (0.06–0.81)	0.024*
<1 year	116	32 (27.6)	1		1	
Anxiety						
Yes	72	39 (54.2)	21.15 (11.08–40.37)	0.001*	13.50 (5.88–31.33)	0.001*
No	378	20 (5.3)	1		1	

## Discussion

In the current study, 16% of transport workers had anxiety and 13% of transport workers had depression according to HADS. Similarly, a cross-sectional study conducted by Taklikar among Navi Mumbai bus drivers found that about 18% and 8% were diagnosed with severe anxiety and depression, respectively [1]. In that study, occupational stress level was moderate among 114 bus drivers and high among four bus drivers [1]. The stress score was significantly high among bus drivers who were older and had a job duration of >10 years [1].

Notably, age, education, marital status, bus route type, years of experience, and depression emerged as significant predictors of anxiety. Whereas type of employment, years of experience, and anxiety were significantly associated with depression. Likewise, a cross-sectional study in China revealed that the mental health of bus drivers was associated with some demographic and personality characteristics [8].

Job factors that contribute to the development of stress in bus drivers were work shift schedules, irregular meal times and poor nutrition, traffic congestion, prolonged period of driving, constant visual and mental alertness, and driving during night hours in bad weather conditions [9]. Bus driving interferes with social support in two ways. The job itself is solitary with little chance for face-to-face contact between co-workers and the work schedule disrupts family and social life [10].

Bus driving is a classic example of a high-strain occupation, with high risks of physical and mental occupational defenselessness, leading to absenteeism and decreased productivity of employees and enterprises [9]. Drivers must respond to multiple unpredicted situations over which they have little control [9]. The main tasks of a bus driver are to drive safely and maintain time schedule [9]. Traffic congestion is another stressor. Typically, stressful jobs are those which have high psychological demands and little decision-making control, in combination with minimum social support on the job [10]. Stress factors may affect the power of coordination of the driver, causing accidents [10]. Consequently, social support helps to protect the individual from experiencing stress.

This study made an attempt to capture the prevalence of mental health issues and their contributing factors among transport workers. To minimize information bias, the questionnaire was developed in alignment with the standard guidelines. In addition, observer bias was reduced as the data collection was done by trained investigators. Non-response rate was minimal owing to good rapport with the transport workers. Nevertheless, as the questionnaire was subjective, errors due to self-reported mental health findings are possible in the present study. The number of days away from home also affects mental health. However, this factor was not considered in this study. The applicability of the results to diverse populations remains ambiguous. Furthermore, it would be beneficial to investigate additional dimensions, such as examining the correlation between anxiety, depression, and cognitive functions.

## Conclusions

Symptoms of anxiety and depression among bus drivers and conductors pose a significant public health challenge. These conditions are detrimental not only to those working within the transport sector but also to the wider public who utilize these services. Unaddressed symptoms can potentially escalate to road traffic accidents, resulting in injuries, disabilities, and even fatalities. Given their vulnerability, transport workers warrant particular attention regarding their mental well-being. Implementing strategies such as job rotation, fostering positive ergonomics, and facilitating social support are promising measures to enhance mental health within this workforce. It is imperative for administrative authorities to take proactive steps for the welfare of transport workers, prioritizing their mental health as a key aspect of occupational safety.
